# Recent advances in understanding the role of phasic dopamine activity

**DOI:** 10.12688/f1000research.19793.1

**Published:** 2019-09-24

**Authors:** Wolfram Schultz

**Affiliations:** 1 Department of Physiology, Development and Neuroscience, University of Cambridge, Cambridge, CB2 3DY, UK

**Keywords:** reward, prediction, learning, aversive, movement

## Abstract

The latest animal neurophysiology has revealed that the dopamine reward prediction error signal drives neuronal learning in addition to behavioral learning and reflects subjective reward representations beyond explicit contingency. The signal complies with formal economic concepts and functions in real-world consumer choice and social interaction. An early response component is influenced by physical impact, reward environment, and novelty but does not fully code prediction error. Some dopamine neurons are activated by aversive stimuli, which may reflect physical stimulus impact or true aversiveness, but they do not seem to code general negative value or aversive prediction error. The reward prediction error signal is complemented by distinct, heterogeneous, smaller and slower changes reflecting sensory and motor contributors to behavioral activation, such as substantial movement (as opposed to precise motor control), reward expectation, spatial choice, vigor, and motivation. The different dopamine signals seem to defy a simple unifying concept and should be distinguished to better understand phasic dopamine functions.

## Introduction

The question “What is dopamine doing?” keeps stubbornly popping up after the discovery of the brain’s dopamine system and its relationships to Parkinson’s disease, psychosis, and drug addiction. Although the efficacy of dopamine receptor–stimulating drugs in alleviating Parkinsonian movement disorders pointed initially to a mere tonic, modulatory role, it became increasingly clear that dopamine is a neurotransmitter not unlike other transmitters and has its own synapses and phasic activity related to stimuli and actions. The ensuing research efforts revealed an amazing array of heterogeneous functions at various time courses and levels of specificity that range from general behavioral activation to precise reward signaling for biological learning, machine learning, and economic choice ^1^. The complexity defies the notion of “one neuronal system equals one function” but likely reflects the workings of an evolutionarily ancient system that governs the individual’s requirements for survival.

This overview describes further conceptual, biological, and economic characterizations of the dopamine reward signal in animals from the past few years, its involvement in social processes, and its distinction from aversive, novelty, sensory, and motor processing. I will follow the notion that the function of an information-processing system can be defined by the relationship of its internal signals to behavior. This knowledge would provide a firm basis for investigating molecular, cellular, and circuit mechanisms. However, detailed descriptions of the recently elucidated fine network properties of dopamine neurons would exceed the topic and limits of this brief review, nor will I be able to discuss molecular signaling, human brain signals, and effects of lesions and systemic dopaminergic drugs that indicate tonic permissive rather than phasic driving influences.

## Further characterization of the reward prediction error signal

Rather than coding rewards and reward-predicting stimuli as they appear in the environment, phasic, sub-second responses in the majority of midbrain dopamine neurons code a reward prediction error. Their activity is increased for one hundred or two hundred milliseconds when a reward or reward-predicting stimulus is better than predicted, their activity is unchanged when these events have the same reward value as their prediction, and their activity is briefly depressed when these events have lower reward value than predicted ^1^.

### Rewarding effect of dopamine neuron stimulation

**Figure 1.**
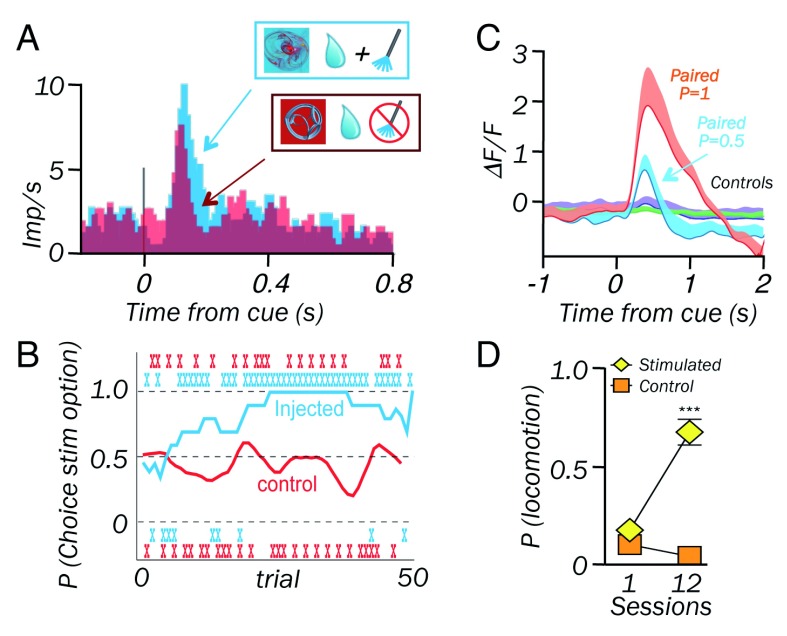
Optogenetic dopamine excitation elicits neuronal learning in dopamine neurons. ( **A**) Increased stimulus response after optogenetic dopamine excitation added to reward (identical juice after each stimulus). Adapted from Stauffer *et al.* ^10^ Figure 5.C, CC BY 4.0. ( **B**) Behavioral learning: gradual increase of choice probability between the two stimuli. Ticks indicate choices in channelrhodopsin-injected animals (blue) and non-injected controls (red). Adapted from Stauffer *et al.* ^10^ Figure 6.B, CC BY 4.0. ( **C**) Graded neuronal learning in rats induced by dopamine excitation at reward time. P = probability of excitation per stimulus appearance ^11^. ( **D**) Behavioral learning: acquisition of locomotion following the stimulus associated with optogenetic excitation ^11^.

Electrical or optical stimulation of dopamine neurons serves as a teaching signal for lever pressing, nose poking, place preference, unblocking, and prevention of extinction ^2– 6^; conversely, optogenetic dopamine inhibition induces place avoidance and behavioral inhibition ^7– 9^. These behavioral effects likely reflect the elicitation of positive and negative reward prediction error signals, respectively. Recent research shows that these behavioral learning functions extend to neuronal learning: monkey dopamine neurons acquire stronger responses to an intrinsically neutral visual stimulus that is followed by optogenetic dopamine stimulation added to juice reward, as compared with a stimulus associated with only that reward ( Figure 1A, B) ^10^. Concomitantly, the animal develops choice preference over 20 to 25 repetitions for the stimulation-associated fractal over an alternative, non-stimulated fractal, even without natural reward. In rats, optogenetic dopamine excitation at the time of reward induces dopamine responses to the stimulus along with driving approach and locomotion ( Figure 1C, D) ^11^. In a further step, dopamine stimulation serves as reward for operantly controlling cortical firing patterns ^12^. These effects together support the hypothesis that bidirectional dopamine reward prediction error responses influence neuronal and behavioral learning.

### Dopamine neurons access reward predictions without explicit association

Standard reward learning paradigms rely on the contingent association with a stimulus, whereas higher learning theories postulate a role for representations beyond explicit reward contingency. Dopamine neurons follow this latter notion ^13^: during sensory preconditioning, two stimuli (A and B) are first presented sequentially. Then reward occurs only with the later stimulus presented alone (B). Then the earlier stimulus (A) is tested for reward prediction. Indeed, dopamine neurons are activated by the test stimulus (A) although it had never been explicitly paired with the reward. Thus, the neurons access a reward representation via the test stimulus (A) that had earlier been associated with the then-unrewarded stimulus (B), defying the simple requirement for direct stimulus–reward contingency.

### Prediction error responses reveal what’s on dopamine’s mind

The reward prediction error response depends on both the reward and the prediction: reward received minus reward predicted. If we know the reward and measure the dopamine response, we can infer the prediction the neuron is accessing.

The idea started with a stimulus sequence that always ends with a reward after a short but random number of steps. A monkey registering only repeated reward omissions would expect progressively less reward, but with experience it would know the reward would come more likely the longer the wait is (increasing hazard rate). Thus, with longer waiting, reward prediction increases and the error when the reward occurs decreases. Indeed, the dopamine response to the reward decreased during waiting, indicating that the neurons accessed the temporally increasing reward prediction derived from the overall task experience (rather than a decreasing prediction derived from the repeating reward omissions) ^14^. A recent experiment confirmed this result in mice but tested also slightly uncertain rewards (probability of *P* = 0.9). Here, the animal never knew for sure whether the reward would ultimately come and might increasingly expect none as time advances (like humans giving up waiting for an unreliable bus). But when the reward does occur, the prediction error and the dopamine response are higher the longer the wait was ^15^. Thus, the dopamine response reflects access to reward predictions that are inferred from the temporal structure of reward probabilities rather than deriving entirely from the occurrence or omission of last rewards. Interestingly, reward-predicting responses in amygdala reflect also temporal reward probability ^16^, indicating that reward neurons in general may access more sophisticated reward representations than hitherto assumed.

Reward predictions accessed by dopamine neurons derive from probability distributions of reward amounts. A larger reward compared with the expected value (predicted mean) of a predicted distribution activates dopamine neurons in monkeys, and a smaller reward induces a depression ^17– 20^. Dopamine responses change their gain depending on the variance of the distribution ^21^, suggesting access to at least the first two statistical moments of distributions. By contrast, with a predicted distribution of only two fixed reward amounts, something unexpected happens in mice: there is no response when either of the two predicted rewards occurs but a graded response in rare probe trials that tends to increase with the absolute difference to each of the two predicted rewards; the response is positive for amounts slightly above the lower reward, negative for amounts slightly below the upper reward, and zero for amounts right between the two rewards ^22^. For an intuitive example, imagine a restaurant with two randomly alternating chefs with widely different ability: when the food is almost but not quite spectacular, we realize the good chef was cooking but may have overlooked something, thus generating a negative prediction error (relative to the predicted superb meal from that chef), even though the food was better than from the other chef and above the mean from both chefs. Thus, dopamine neurons access rich reward probability distributions via their statistical moments but can access individual elements when distributions are very restricted. As seen during waiting ^14, 15^ and reward reversal ^23^, the reward predictions accessed by dopamine neurons derive not only from recent rewards but also from the overall reward structure of the environment.

Perceptual choices help to further reveal what’s on dopamine’s mind. Dopamine responses to a set of choice options reflect the animal’s future choice. When a monkey chooses the more frequently rewarded option, the stimulus response is stronger compared with choosing the less often rewarded option, despite identical option presentation. As reward probability constitutes value, the neurons code “chosen value” (that is, the value of the option the animal chooses) rather than the mean value of all options ^24, 25^. The chosen value response occurs to the stimulus and partly precedes and thus predicts the choice. In these straightforward tests, the animal chooses, with some stochasticity, between values that are firmly associated with the options. By contrast, in perceptual random-dot motion choice tasks, the value depends on the animal’s discrimination of motion direction, and the reward probabilities are not firmly associated with constant, unequivocally marked options. Higher motion coherence allows better discrimination and thus increases the probability of getting a reward. Thus, with higher coherence, reward value increases monotonically when choosing the correct motion direction but decreases monotonically when choosing the opposite, incorrect direction. Dopamine neurons in monkeys and mice show exactly this graded chosen value response during random-dot motion and contrast detection tasks ^26, 27^. The value responses before each choice derive from the combination of the animal’s stimulus assessment and the subjective probability of making a correct discrimination (“subjective” in the sense of perception rather than individual economic probability weighing). As the targets are not distinctly marked for value, the responses cannot simply reflect the experienced reward probability for a given target.

Taken together, dopamine neurons have access to representations of future rewards that not only are associated with explicit stimuli but also derive from environmental factors like context, task structure, and time. These internal representations may be more globally called belief states and, when they reflect prior probabilities, Bayesian belief states ^22, 26^. These representations or beliefs are parts of reward predictions that affect dopamine neurons, which report their deviation from the actual obtained primary and conditioned rewards as “reward prediction error”.

### Neuroeconomics

Rewards don’t exist; they are made up by our minds. The third steak during a dinner is not attractive although it is pretty similar to the first two appetizing steaks. Plenty of other examples confirm that reward value is subjective and depends on non-physical factors like satiety, delay, and risk. While we can forever test individual cases of subjective value, economic theory provides concepts for understanding subjective value and preferences and predicting behavioral choices under various conditions, including risk. An example is the utility signal of dopamine neurons that transcends the ad-hoc coding of subjective value ^19^. This neuronal result aligns biological reward to economic choice and constitutes a prerequisite for understanding how individuals maximize utility for momentary and evolutionary benefit.

**Figure 2.**
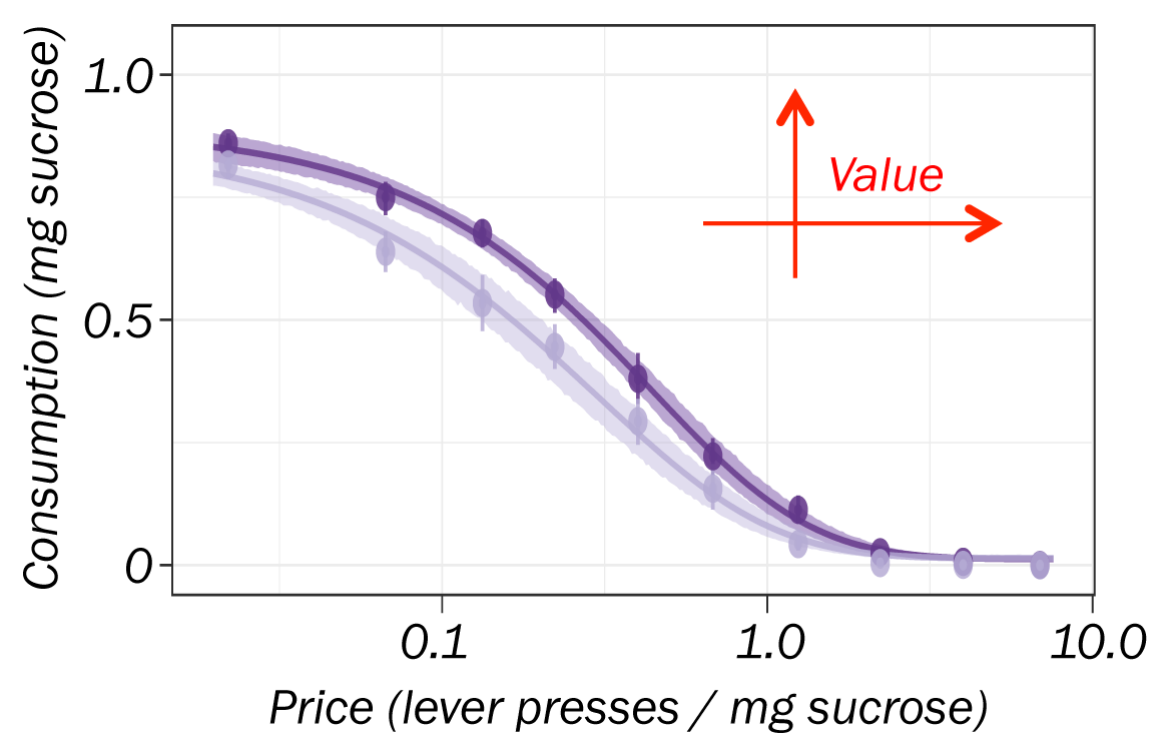
Optogenetic dopamine stimulation enhances consumer value. Dopamine stimulation induces rats to consume more sucrose for the same price (lever press per pellet, vertical arrow) and maintain the same consumption despite increasing price (horizontal arrows), reflecting the value increase by the stimulation-elicited positive reward prediction error signal. Light blue: without stimulation; dark blue: with stimulation at reward time ^28^.

But what would a dopamine signal for such a theoretical decision variable do in a real-world scenario? One of the most intuitive and reliable phenomena in economics is the price–demand relationship. As the price goes up, consumption goes down; people buy less stuff when it gets more expensive. But if the good becomes more valuable, demand increases, which shifts price–demand curves to the right. Price can be modeled as number of lever presses in rats, and value can be enhanced by dopamine stimulation, although further known factors affecting consumption may be too extensive for an initial, well-controlled study, such as availability of alternatives, time, and effort. How then would a dopamine economic value (utility) signal affect consumer choice? Indeed, inducing a positive dopamine reward prediction error signal by optogenetic excitation at the reward shifts the curves upward and rightward, indicating that the stimulation enhances value, thereby increasing demand at same price and maintaining same consumption despite higher price ( Figure 2) ^28^. Stimulation at the reward-predicting cue has the opposite effect (by lowering reward value due to a negative prediction error elicited by the reward following the enhanced value prediction). This well-conceptualized situation, even with the restrictions imposed on an initial study, demonstrates that the dopamine utility signal has a very practical application; it affects daily consumer choice by influencing the value of a good. This beautiful result, outside the beaten path, suggests many follow-up experiments.

### Social settings: valuing own and other’s reward

Rewards are fine for me but may not be so great when somebody else receives them instead of me. Monkeys see it the same way; they value rewards more when they occur more frequently for themselves but not so much when they occur for another monkey, as shown by licking and binary choice. Dopamine neurons follow this social reward valuation; higher probability of own water reward elicits stronger responses, confirming standard reward value coding, whereas higher reward probability for the other monkey reduces own dopamine responses ^29^. It seems that this disadvantageous reward inequity has negative reward value for dopamine neurons. Thus, dopamine neurons register everybody’s rewards but value them only relative to their host. Their primary concern with own reward resembles that of most reward neurons in the striatum ^30^, some of which sense disadvantageous reward inequity ^31^.

## The dopamine prediction error signal: purely reward?

### A response that is only a component

Environmental rewards and reward-predicting stimuli contain a non-value component that impacts on sensory receptors, but their identification and evaluation take a few tens or hundreds of milliseconds. Dopamine neurons, in analogy to other neuronal systems, show an early unselective activation, which reflects sensory detection of the stimulus ^32^ and constitutes a default signal for any potential reward in the environment; it is quickly replaced, before any behavioral action, by the subsequent prediction error component that codes reward value ^19, 33– 35^; recent studies confirm this notion ^36^. Thus, the initial, non-reward activation constitutes an integral part of the dopamine reward response. Its identification requires temporal resolution in the ten-millisecond range and is often difficult, in particular with unrewarded, value-less stimuli not allowing independent variation of sensory and reward parameters.

**Figure 3.**
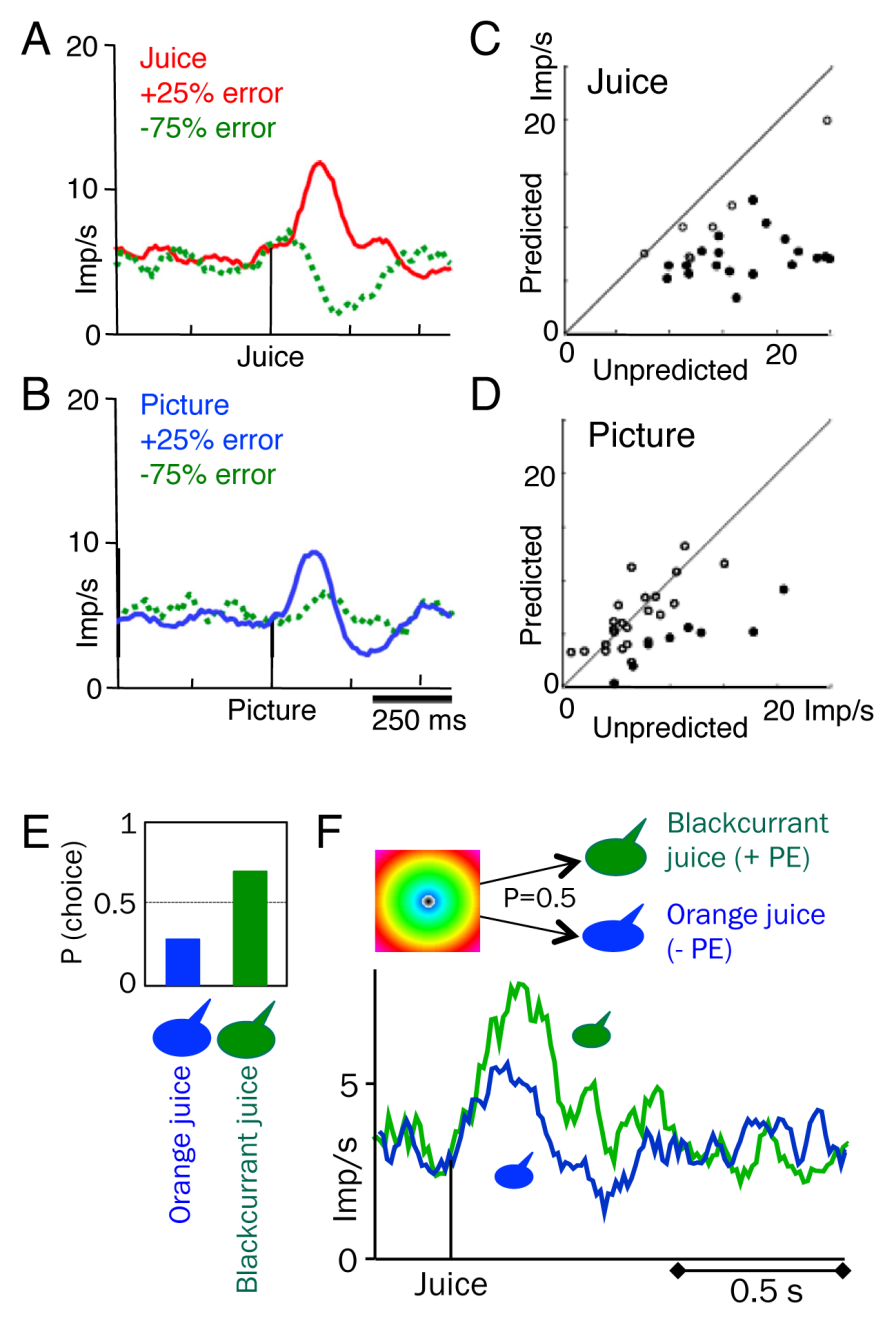
Surprise salience coding with non-rewarding stimuli contrasts with reward prediction error coding ( **A**) Bidirectional prediction error coding for juice reward. The animal received juice reward in 75% of trials but not in 25% of trials. Hence, a reward that did occur generated a 25% positive prediction error, and an omitted reward generated a 75% negative prediction error. ( **B**) With similar 75 to 25% presentation of non-rewarding arbitrary (fractal) picture, unidirectional response enhancement with surprising picture occurrence (+25% picture prediction error), without negative error coding with picture omission (−75% picture error). ( **C**) Reward response increase with unpredicted reward delivery (compatible with positive reward prediction error coding). Closed circles indicate significant differences ( *P* <0.05; *t* test). ( **D**) Smaller response enhancement with unpredicted picture occurrence, reflecting surprise salience. **A**– **D** are reused from Kobayashi and Schultz ^38^ Figure 4 (A, B, E, F), CC BY 3.0. ( **E**) Preference for blackcurrant over orange juice in binary, simultaneous choice (same liquid amounts), indicating higher value of blackcurrant than orange juice ^18^. ( **F**) Dopamine prediction error response for juice identity reflects reward value. The concentric stimulus predicts equiprobable delivery of either blackcurrant or orange juice; the neuronal response reflects the prediction error between the value of the received juice and the stimulus-predicted mean value of the two juices (green: positive; blue: negative, with initial-component activation) ^18^.

Several factors affect the initial, sensory dopamine activation. First, it increases with physical impact and salience, irrespective of reward or aversive value ^34^. Second, it is elicited and enhanced by neutral or punishment-predicting stimuli that resemble rewards or occur in rewarding contexts ^37– 39^. Finally, it occurs with novel stimuli in humans, monkeys, and mice ^25, 40– 42^. The novelty component decays during conditioning (due to repetition), whereas the reward-predicting component increases ^25, 42^. The unpredicted occurrence of an unrewarded picture and positive sensory prediction errors enhance the initial-component response but, in contrast to bidirectional reward prediction error coding, picture omission does not seem to elicit a dopamine depression in monkeys and rats ^33, 38, 43^ ( Figure 3A–D). Thus, the initial dopamine response component seems to code surprise salience rather than a full, bidirectional prediction error. In contrast to the initial sensory component, delivery of different juices with different sensory attributes elicits a bidirectional reward prediction error response that reflects the value of the juices ( Figure 3E, F).

### Aversive responses

For 40 years, many studies, including our own, reported activations by aversive stimuli in some dopamine neurons (for references, see 35). However, aversive events contain several components, as do rewards, and their dissociation concluded that dopamine activations by aversive stimuli reflect physical impact (first component) rather than aversiveness ^34^; aversiveness is coded not at all ^34^ or as depression of activity reflecting negative reward value (second component) ^44, 45^. Dopamine reward neurons are also activated by negative punishment prediction error, which has positive value (double negative) ^39, 45, 46^, by rebound from aversive depression ^34, 45^, and by prediction of relief from punishment ^45– 47^, which is rewarding ^48, 49^. Thus, some of the recently reported activations by aversive air puff, sound, and foot shock ^44, 45^ might reflect rewarding relief from the threat these stimuli might pose to the animals, even if these neurons do not code standard reward.

In contrast to these reward responses, recent studies report activations in dopamine subgroups in lateral substantia nigra, striatum tail, and ventro-medial nucleus accumbens shell in response to air puff, intense sound, and foot shock but not with physically less intense aversive quinine nor much with reward ^42, 44, 45^. These responses may reflect physical impact or aversion or both. The foot shock activation transfers to predictive stimuli during learning in ventro-medial nucleus accumbens shell ^45^. This result would refute a possible relation to physical impact, which is unchanged, but it might also reflect temporal surprise salience; it might even indicate transfer of an early-component sensory impact response in analogy to the known transfer of the subsequent value component. Nonetheless, these neurons differ in molecular and physiological properties and have striatal projection territories different from those of the typical, straightforward reward-processing dopamine neurons ^44, 45^. Foot shock omission fails to elicit depressions in these dopamine neurons ^45^; this lack of bidirectional prediction error coding would make an involvement in reinforcement learning less direct. Furthermore, optogenetic excitation of dopamine axons in striatum tail elicits behavioral aversion ^44^, indicating a truly aversive function (though without completely mimicking the brain’s mechanics of natural excitation). The physically less intense quinine is ineffective despite its behavioral aversiveness ^44^, which argues for a contribution of physical impact and against general negative value coding.

Thus, if physical impact remains an option for explaining activations by aversive stimuli, we might be dealing with the opposite tails of two continuous probability distributions: one for physical impact and one for value. Then dopamine neurons with activations by aversive stimuli might lie at the high end of the physical impact distribution, and their weak reward coding would be at the low end of the value distribution. On the other hand, despite all the caveats, optogenetics may have uncovered groups of dopamine neurons that are truly activated by specific punishers and thus differ qualitatively from reward-processing dopamine neurons ^45^, after 40 years of trying to nail them. If so, they might be parts of an ancient system detecting fear (of air puff, intense sound, foot shock, and novelty) rather than disgust (quinine) ^44^ and contrast with the abundant reward-coding dopamine neurons that are depressed by aversive stimuli and code outcome value monotonically from negative to positive ^39, 44^. Dopamine neurons in fruit flies show similar response diversity—about 130 neurons code reward and 12 neurons code punishment ^50^ suggesting preservation across a huge evolutionary range. So, ten years from now, will we know whether the dopamine activations by aversive stimuli reflect physical impact or aversiveness or maybe both?

## Behavioral activation

Even though the common assumption of one brain system equals one function may not hold for dopamine ^1^, such multifunctionality seems perplexing and gives rise to the question “What is dopamine doing?”

### Movement or not movement

**Figure 4.**
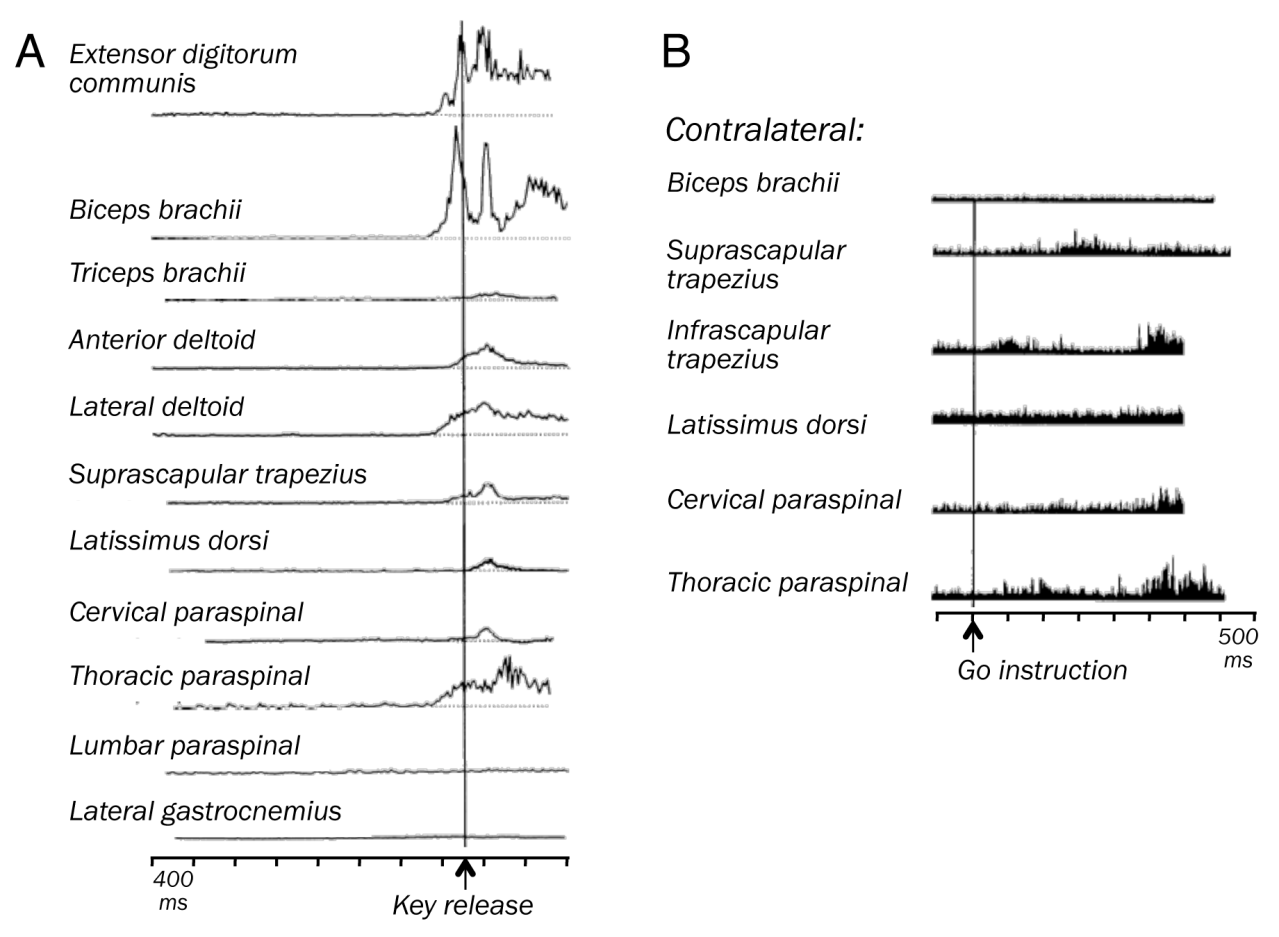
Monitoring of muscle activation during unilateral reaching in macaque monkeys ( **A**) Activity in forearm, upper arm, shoulder, back, and leg muscles during self-initiated arm movements (rectified electromyograms). Some midbrain dopamine neurons show slow activations before (12%) and during (16%) these movements. From Romo and Schultz ^55^. ( **B**) Activity even in contralateral muscles during instructed, stimulus-triggered arm reaching movements. From Schultz and Romo ^70^ with permission from Springer Nature, RightsLink License Number: 4662430242555.

The earliest behavioral studies of midbrain dopamine neurons and striatal dopamine concentrations in monkeys and rats report heterogeneous activations and depressions for a second or more with movements ^51– 55^. Dopamine changes are associated with task events such as large contralateral or ipsilateral arm reaching movements (16–44% and 15–17% of neurons, respectively), self-initiated arm movements (12%), reward delivery and mouth movements (9%), and full trial duration (5%). However, such changes are absent with more concise movements, such as well-controlled arm flexion-extension ^56^, stereotyped reaching ^41^, sluggish reaching elicited by offset of a stimulus ^57^, and spontaneous and stimulus-driven eye movements ^57^. The monitoring of large numbers of individual muscles in monkeys ( Figure 4) shows that these heterogeneous dopamine changes are unrelated to specific movements or motor control but reflect the behavioral activation underlying large movements, derived from the activity of many muscles ^55, 57, 58^ and of sensory receptors in muscle, joint, and skin associated with such movements, a global process that might also be called vigor or even motivation.

### Movement activation

The advent of dopamine voltammetry, molecular identification, optogenetics, and optical recording allows us to further characterize these behavior-related changes, associate them with different neuronal populations and their projection territories, and distinguish them from reward prediction error responses. Recent studies describe dopamine changes when rodents move in open fields, small chambers, levers, nose poke ports, T-mazes, running wheels, and trackballs ^6, 59– 68^, whereas specific motor processes engaging only few muscles are ineffective ^69^. The dopamine changes are heterogeneous in terms of timing during test trials, behavioral variable being encoded, and midbrain location. Thus, early in each trial, activity in distinct dopamine neurons varies with different movement parameters like speed and acceleration, whereas at trial end more neurons code mouth movement or reward ^68^. While some studies provide fine-grained statistical dissociation ^68^, some of the effective behavioral variables, like reward expectation leading to faster movement and movement speed reflecting vigor and motivation, might be intercorrelated; indeed, a common variable underlying these behaviors might be arousal and general behavioral activation. The molecular, cellular, and input heterogeneity of dopamine neuron groups and the differential projection topography between midbrain and striatum ^71– 73^ would allow specific dopamine influences on particular postsynaptic targets. Correspondingly, optogenetic dopamine excitation elicits locomotion and biases choice depending on the midbrain region being stimulated, whereas inhibition elicits opposite effects ^61, 64, 65^, suggesting an active behavioral role of the observed dopamine changes (even without knowing the animal’s “feeling” when receiving a dopamine shock without accompanying sensory or motor cortex activity). By contrast, some motivation-related changes in striatal dopamine concentration are not associated with dopamine impulse changes in the soma ^67^ and may derive from local presynaptic influences that have long been recognized ^74, 75^. (As with other neurotransmitter systems, dopamine function depends on transmitter release and postsynaptic receptors in addition to the temporally precise impulse responses.)

### Comparison with reward prediction error coding

The amazing spectrum and heterogeneity of dopamine relationships to behavioral activation contrast with the rather stereotyped reward prediction error response that varies across neurons in only a single scalar parameter ^36^. The prediction error response stands out more; it is more phasic and has a higher instantaneous impulse rate and a shorter duration than the changes related to behavioral activation. These differences are particularly evident with the high temporal resolution of neurophysiological impulse responses. Nevertheless, the detection of prediction error responses requires explicit events that allow to identify predictions and to subtract their value from that of the reward. Analyses using reinforcement models help to further identify dopamine prediction error responses in elaborate tasks ^64, 76^.

How might these seemingly separate modes of dopamine action relate to each other? Despite attempts to derive a common activational role ^77^, it is currently unclear how the heterogeneous relationships to behavioral activation might emerge from prediction error coding. One may dissociate the behavioral activation from prediction error coding by their respective spatial and non-spatial specificity ^78^ or explain the dopamine voltammetry signal during movement and reward expectation by prediction error coding ^79– 81^, or behavioral activation and reward prediction error might be coded in different dopamine groups. In rodents, movement relationships are more frequent in substantia nigra dopamine neurons and their striatum-projecting regions, whereas reward prediction error coding is abundant in ventral tegmental area neurons and their nucleus accumbens projection ^6, 62, 67, 68^. These differences are gradual and do not constitute the strong medio-lateral midbrain or the ventro-dorsal striatum dichotomy seen in regional lesion experiments. Similar graded, rather than strict, differences are seen in monkeys, whose dopamine neurons in substantia nigra signal reward less frequently (<60%) than in ventral tegmental area (>70–80%) ^41, 82^; in corresponding striatal projection territories, reward expectation affects 40 to 50% of caudate and anterior putamen neurons and more than 75% of nucleus accumbens neurons ^83^.

### Multiple dopamine functions

Thus, the notion of one neuronal system having exactly one function may not be valid for dopamine neurons, however hard we try. Maybe such an evolutionarily ancient system, which exists already in fruit flies, has multiple functions that are difficult to capture in a single term. A common denominator for the role of phasic dopamine activity might be to get the animal what it needs to survive, like detecting reward and coding the action for obtaining it (the two key components of motivation), although that sounds awfully superficial given the intricate complexity of the system.

## The future

The investigation of dopamine function and the underlying networks are currently in full swing. The past several years have revealed many details that help us get a better understanding of dopamine function, and lots of mysticism has disappeared. We are not dealing with a system with clear-cut and well-parcellated functions, but we know that some of the dopamine functions are crucial for the animal’s survival. What we don’t know are at least two things.

How does the dopamine reward signal, as the strongest component of dopamine function, get us the best reward and thus help evolutionary fitness? An obvious approach is to study economic decision-making, which has well-developed concepts for maximizing utility. This approach assumes that decision makers identify, process, and deliberate about all available options and have clear preferences, which underlies the first Von Neumann–Morgenstern utility axiom (“completeness”). But there are many exceptions to rational decision-making, and many decisions are not based on identifiable options. We often just do what we do without actively considering the alternatives. What is the role of dopamine neurons in these processes?

As the investigation of dopamine function has revealed a number of important processes, then what are the other “neuromodulatory” systems hiding? Can we get a handle on norepinephrine after its attentional functions have been so well described ^84^? And what about serotonin— would it have several, diverse functions ^85, 86^ but ultimately a coherent denominator? And what about acetylcholine? We have tons of work to do.

Of course, all of these processes may go wrong in brain disorders, which affect more than 20% of the population and present a major human challenge. For that reason, we should invest substantial portions of our wealth into all fields of neuroscience.

## References

[1] Schultz W: Multiple dopamine functions at different time courses. *Annu Rev Neurosci.* 2007;30:259–88. 10.1146/annurev.neuro.28.061604.135722 17600522

[2] Corbett D Wise RA: Intracranial self-stimulation in relation to the ascending dopaminergic systems of the midbrain: a moveable electrode mapping study. *Brain Res.* 1980;185(1):1–15. 10.1016/0006-8993(80)90666-6 7353169

[3] Tsai HC Zhang F Adamantidis A: Phasic firing in dopaminergic neurons is sufficient for behavioral conditioning. *Science.* 2009;324(5930):1080–4. 10.1126/science.1168878 19389999 PMC5262197

[4] Kim KM Baratta MV Yang A: Optogenetic mimicry of the transient activation of dopamine neurons by natural reward is sufficient for operant reinforcement. *PLoS One.* 2012;7(4):e33612. 10.1371/journal.pone.0033612 22506004 PMC3323614

[5] Steinberg EE Keiflin R Boivin JR: A causal link between prediction errors, dopamine neurons and learning. *Nat Neurosci.* 2013;16(7):966–73. 10.1038/nn.3413 23708143 PMC3705924

[6] Coddington LT Dudman JT: The timing of action determines reward prediction signals in identified midbrain dopamine neurons. *Nat Neurosci.* 2018;21(11):1563–73. 10.1038/s41593-018-0245-7 30323275 PMC6226028

[7] Tan KR Yvon C Turiault M: GABA neurons of the VTA drive conditioned place aversion. *Neuron.* 2012;73(6):1173–83. 10.1016/j.neuron.2012.02.015 22445344 PMC6690362

[8] Chang CY Esber GR Marrero-Garcia Y: Brief optogenetic inhibition of dopamine neurons mimics endogenous negative reward prediction errors. *Nat Neurosci.* 2016;19(1):111–6. 10.1038/nn.4191 26642092 PMC4696902

[9] Chang CY Gardner MPH Conroy JC: Brief, But Not Prolonged, Pauses in the Firing of Midbrain Dopamine Neurons Are Sufficient to Produce a Conditioned Inhibitor. *J Neurosci.* 2018;38(41):8822–30. 10.1523/JNEUROSCI.0144-18.2018 30181136 PMC6181314

[10] Stauffer WR Lak A Yang A: Dopamine Neuron-Specific Optogenetic Stimulation in Rhesus Macaques. *Cell.* 2016;166(6):1564–1571.e6. 10.1016/j.cell.2016.08.024 27610576 PMC5018252

[11] Saunders BT Richard JM Margolis EB: Dopamine neurons create Pavlovian conditioned stimuli with circuit-defined motivational properties. *Nat Neurosci.* 2018;21(8):1072–83. 10.1038/s41593-018-0191-4 30038277 PMC6082399

[12] Athalye VR Santos FJ Carmena JM: Evidence for a neural law of effect. *Science.* 2018;359(6379):1024–9. 10.1126/science.aao6058 29496877

[13] Sadacca BF Jones JL Schoenbaum G: Midbrain dopamine neurons compute inferred and cached value prediction errors in a common framework. *eLife.* 2016;5: pii: e13665. 10.7554/eLife.13665 26949249 PMC4805544

[14] Nakahara H Itoh H Kawagoe R: Dopamine neurons can represent context-dependent prediction error. *Neuron.* 2004;41(2):269–80. 10.1016/s0896-6273(03)00869-9 14741107

[15] Starkweather CK Babayan BM Uchida N: Dopamine reward prediction errors reflect hidden-state inference across time. *Nat Neurosci.* 2017;20(4):581–9. 10.1038/nn.4520 28263301 PMC5374025

[16] Bermudez MA Göbel C Schultz W: Sensitivity to temporal reward structure in amygdala neurons. *Curr Biol.* 2012;22(19):1839–44. 10.1016/j.cub.2012.07.062 22959346 PMC3526777

[17] Fiorillo CD Tobler PN Schultz W: Discrete coding of reward probability and uncertainty by dopamine neurons. *Science.* 2003;299(5614):1898–902. 10.1126/science.1077349 12649484

[18] Lak A Stauffer WR Schultz W: Dopamine prediction error responses integrate subjective value from different reward dimensions. *Proc Natl Acad Sci U S A.* 2014;111(6):2343–8. 10.1073/pnas.1321596111 24453218 PMC3926061

[19] Stauffer WR Lak A Schultz W: Dopamine reward prediction error responses reflect marginal utility. *Curr Biol.* 2014;24(21):2491–500. 10.1016/j.cub.2014.08.064 25283778 PMC4228052

[20] Tian J Uchida N: Habenula Lesions Reveal that Multiple Mechanisms Underlie Dopamine Prediction Errors. *Neuron.* 2015;87(6):1304–16. 10.1016/j.neuron.2015.08.028 26365765 PMC4583356

[21] Tobler PN Fiorillo CD Schultz W: Adaptive coding of reward value by dopamine neurons. *Science.* 2005;307(5715):1642–5. 10.1126/science.1105370 15761155

[22] Babayan BM Uchida N Gershman SJ: Belief state representation in the dopamine system. *Nat Commun.* 2018;9(1):1891. 10.1038/s41467-018-04397-0 29760401 PMC5951832

[23] Bromberg-Martin ES Matsumoto M Hong S: A pallidus-habenula-dopamine pathway signals inferred stimulus values. *J Neurophysiol.* 2010;104(2):1068–76. 10.1152/jn.00158.2010 20538770 PMC2934919

[24] Morris G Nevet A Arkadir D: Midbrain dopamine neurons encode decisions for future action. *Nat Neurosci.* 2006;9(8):1057–63. 10.1038/nn1743 16862149

[25] Lak A Stauffer WR Schultz W: Dopamine neurons learn relative chosen value from probabilistic rewards. *eLife.* 2016;5: pii: e18044. 10.7554/eLife.18044 27787196 PMC5116238

[26] Lak A Nomoto K Keramati M: Midbrain Dopamine Neurons Signal Belief in Choice Accuracy during a Perceptual Decision. *Curr Biol.* 2017;27(6):821–32. 10.1016/j.cub.2017.02.026 28285994 PMC5819757

[27] Lak A Okun M Moss M: Neural basis of learning guided by sensory confidence and reward value. *bioRxiv.* 2018 10.1101/411413

[28] Schelp SA Pultorak KJ Rakowski DR: A transient dopamine signal encodes subjective value and causally influences demand in an economic context. *Proc Natl Acad Sci U S A.* 2017;114(52):E11303–E11312. 10.1073/pnas.1706969114 29109253 PMC5748169

[29] Noritake A Ninomiya T Isoda M: Social reward monitoring and valuation in the macaque brain. *Nat Neurosci.* 2018;21(10):1452–62. 10.1038/s41593-018-0229-7 30224807

[30] Baez-Mendoza R Harris CJ Schultz W: Activity of striatal neurons reflects social action and own reward. *Proc Natl Acad Sci U S A.* 2013;110(41):16634–9. 10.1073/pnas.1211342110 24062436 PMC3799314

[31] Báez-Mendoza R van Coeverden CR Schultz W: A neuronal reward inequity signal in primate striatum. *J Neurophysiol.* 2016;115(1):68–79. 10.1152/jn.00321.2015 26378202 PMC4760476

[32] Dommett E Coizet V Blaha CD: How visual stimuli activate dopaminergic neurons at short latency. *Science.* 2005;307(5714):1476–9. 10.1126/science.1107026 15746431

[33] Nomoto K Schultz W Watanabe T: Temporally extended dopamine responses to perceptually demanding reward-predictive stimuli. *J Neurosci.* 2010;30(32):10692–702. 10.1523/JNEUROSCI.4828-09.2010 20702700 PMC3297489

[34] Fiorillo CD Song MR Yun SR: Multiphasic temporal dynamics in responses of midbrain dopamine neurons to appetitive and aversive stimuli. *J Neurosci.* 2013;33(11):4710–25. 10.1523/JNEUROSCI.3883-12.2013 23486944 PMC3873404

[35] Schultz W: Dopamine reward prediction-error signalling: a two-component response. *Nat Rev Neurosci.* 2016;17(3):183–95. 10.1038/nrn.2015.26 26865020 PMC5549862

[36] Eshel N Tian J Bukwich M: Dopamine neurons share common response function for reward prediction error. *Nat Neurosci.* 2016;19(3):479–86. 10.1038/nn.4239 26854803 PMC4767554

[37] Mirenowicz J Schultz W: Preferential activation of midbrain dopamine neurons by appetitive rather than aversive stimuli. *Nature.* 1996;379(6564):449–51. 10.1038/379449a0 8559249

[38] Kobayashi S Schultz W: Reward contexts extend dopamine signals to unrewarded stimuli. *Curr Biol.* 2014;24(1):56–62. 10.1016/j.cub.2013.10.061 24332545 PMC3898276

[39] Matsumoto H Tian J Uchida N: Midbrain dopamine neurons signal aversion in a reward-context-dependent manner. *eLife.* 2016;5: pii: e17328. 10.7554/eLife.17328 27760002 PMC5070948

[40] Kamiński J Mamelak AN Birch K: Novelty-Sensitive Dopaminergic Neurons in the Human Substantia Nigra Predict Success of Declarative Memory Formation. *Curr Biol.* 2018;28(9):1333–1343.e4. 10.1016/j.cub.2018.03.024 29657115 PMC5973539

[41] Ljungberg T Apicella P Schultz W: Responses of monkey dopamine neurons during learning of behavioral reactions. *J Neurophysiol.* 1992;67(1):145–63. 10.1152/jn.1992.67.1.145 1552316

[42] Menegas W Babayan BM Uchida N: Opposite initialization to novel cues in dopamine signaling in ventral and posterior striatum in mice. *eLife.* 2017;6: pii: e21886. 10.7554/eLife.21886 28054919 PMC5271609

[43] Takahashi YK Batchelor HM Liu B: Dopamine Neurons Respond to Errors in the Prediction of Sensory Features of Expected Rewards. *Neuron.* 2017;95(6):1395–1405.e3. 10.1016/j.neuron.2017.08.025 28910622 PMC5658021

[44] Menegas W Akiti K Amo R: Dopamine neurons projecting to the posterior striatum reinforce avoidance of threatening stimuli. *Nat Neurosci.* 2018;21(10):1421–30. 10.1038/s41593-018-0222-1 30177795 PMC6160326

[45] de Jong JW Afjei SA Pollak Dorocic I: A Neural Circuit Mechanism for Encoding Aversive Stimuli in the Mesolimbic Dopamine System. *Neuron.* 2019;101(1):133–151.e7. 10.1016/j.neuron.2018.11.005 30503173 PMC6317997

[46] Salinas-Hernández XI Vogel P Betz S: Dopamine neurons drive fear extinction learning by signaling the omission of expected aversive outcomes. *eLife.* 2018;7: pii: e38818. 10.7554/eLife.38818 30421719 PMC6257816

[47] Oleson EB Gentry RN Chioma VC: Subsecond dopamine release in the nucleus accumbens predicts conditioned punishment and its successful avoidance. *J Neurosci.* 2012;32(42):14804–8. 10.1523/JNEUROSCI.3087-12.2012 23077064 PMC3498047

[48] Solomon RL Corbit JD: An opponent-process theory of motivation. I. Temporal dynamics of affect. *Psychol Rev.* 1974;81(2):119–45. 10.1037/h0036128 4817611

[49] Gerber B Yarali A Diegelmann S: Pain-relief learning in flies, rats, and man: Basic research and applied perspectives. *Learn Mem.* 2014;21(4):232–52. 10.1101/lm.032995.113 24643725 PMC3966540

[50] Waddell S: Reinforcement signalling in *Drosophila*; dopamine does it all after all. *Curr Opin Neurobiol.* 2013;23(3):324–9. 10.1016/j.conb.2013.01.005 23391527 PMC3887340

[51] Schultz W Ruffieux A Aebischer P: The activity of pars compacta neurons of the monkey substantia nigra in relation to motor activation. *Exp Brain Res.* 1983;51(3):377–387. 10.1007/BF00237874

[52] Lindsay WS Herndon JG Jr Blakely RD: Voltammetric recording from neostriatum of behaving rhesus monkey. *Brain Res.* 1981;220(2):391–6. 10.1016/0006-8993(81)91231-2 7284764

[53] Freed CR Yamamoto BK: Regional brain dopamine metabolism: a marker for the speed, direction, and posture of moving animals. *Science.* 1985;229(4708):62–5. 10.1126/science.4012312 4012312

[54] Schultz W: Responses of midbrain dopamine neurons to behavioral trigger stimuli in the monkey. *J Neurophysiol.* 1986;56(5):1439–61. 10.1152/jn.1986.56.5.1439 3794777

[55] Romo R Schultz W: Dopamine neurons of the monkey midbrain: Contingencies of responses to active touch during self-initiated arm movements. *J Neurophysiol.* 1990;63(3):592–606. 10.1152/jn.1990.63.3.592 2329363

[56] DeLong MR Crutcher MD Georgopoulos AP: Relations between movement and single cell discharge in the substantia nigra of the behaving monkey. *J Neurosci.* 1983;3(8):1599–606. 10.1523/JNEUROSCI.03-08-01599.1983 6875659 PMC6564529

[57] Schultz W Romo R: Dopamine neurons of the monkey midbrain: contingencies of responses to stimuli eliciting immediate behavioral reactions. *J Neurophysiol.* 1990;63(3):607–24. 10.1152/jn.1990.63.3.607 2329364

[58] Hassani OK Cromwell HC Schultz W: Influence of Expectation of Different Rewards on Behavior-Related Neuronal Activity in the Striatum. *J Neurophysiol.* 2001;85(6):2477–89. 10.1152/jn.2001.85.6.2477 11387394

[59] Roitman MF Stuber GD Phillips PE: Dopamine operates as a subsecond modulator of food seeking. *J Neurosci.* 2004;24(6):1265–71. 10.1523/JNEUROSCI.3823-03.2004 14960596 PMC6730321

[60] Howe MW Tierney PL Sandberg SG: Prolonged dopamine signalling in striatum signals proximity and value of distant rewards. *Nature.* 2013;500(7464):575–9. 10.1038/nature12475 23913271 PMC3927840

[61] Howe MW Dombeck DA: Rapid signalling in distinct dopaminergic axons during locomotion and reward. *Nature.* 2016;535(7613):505–10. 10.1038/nature18942 27398617 PMC4970879

[62] Parker NF Cameron CM Taliaferro JP: Reward and choice encoding in terminals of midbrain dopamine neurons depends on striatal target. *Nat Neurosci.* 2016;19(6):845–54. 10.1038/nn.4287 27110917 PMC4882228

[63] Dodson PD Dreyer JK Jennings KA: Representation of spontaneous movement by dopaminergic neurons is cell-type selective and disrupted in parkinsonism. *Proc Natl Acad Sci U S A.* 2016;113(15):E2180–E2188. 10.1073/pnas.1515941113 27001837 PMC4839395

[64] Hamid AA Pettibone JR Mabrouk OS: Mesolimbic dopamine signals the value of work. *Nat Neurosci.* 2016;19(1):117–26. 10.1038/nn.4173 26595651 PMC4696912

[65] da Silva JA Tecuapetla F Paixão V: Dopamine neuron activity before action initiation gates and invigorates future movements. *Nature.* 2018;554(7691):244–8. 10.1038/nature25457 29420469

[66] Kremer Y Flakowski J Rohner C: VTA dopamine neurons multiplex external with internal representations of goal-directed action. *bioRxiv.* 2018 10.1101/408062

[67] Mohebi A Pettibone JR Hamid AA: Dissociable dopamine dynamics for learning and motivation. *Nature.* 2019;570(7759):65–70. 10.1038/s41586-019-1235-y 31118513 PMC6555489

[68] Engelhard B Finkelstein J Cox J: Specialized coding of sensory, motor and cognitive variables in VTA dopamine neurons. *Nature.* 2019;570(7762):509–13. 10.1038/s41586-019-1261-9 31142844 PMC7147811

[69] Cohen JY Haesler S Vong L: Neuron-type-specific signals for reward and punishment in the ventral tegmental area. *Nature.* 2012;482(7383):85–8. 10.1038/nature10754 22258508 PMC3271183

[70] Schultz W Romo R: Role of primate basal ganglia and frontal cortex in the internal generation of movements. I. Preparatory activity in the anterior striatum. *Exp Brain Res.* 1992;91(3):363–84. 10.1007/bf00227834 1483512

[71] Lammel S Hetzel A Häckel O: Unique Properties of Mesoprefrontal Neurons within a Dual Mesocorticolimbic Dopamine System. *Neuron.* 2008;57(5):760–73. 10.1016/j.neuron.2008.01.022 18341995

[72] Beier KT Steinberg EE DeLoach KE: Circuit Architecture of VTA Dopamine Neurons Revealed by Systematic Input-Output Mapping. *Cell.* 2015;162(3):622–34. 10.1016/j.cell.2015.07.015 26232228 PMC4522312

[73] Morales M Margolis EB: Ventral tegmental area: cellular heterogeneity, connectivity and behaviour. *Nat Rev Neurosci.* 2017;18(2):73–85. 10.1038/nrn.2016.165 28053327

[74] Glowinski J Chéramy A Romo R: Presynaptic regulation of dopaminergic transmission in the striatum. *Cell Mol Neurobiol.* 1988;8(1):7–17. 10.1007/BF00712906 2900072 PMC11567314

[75] Threlfell S Lalic T Platt NJ: Striatal dopamine release is triggered by synchronized activity in cholinergic interneurons. *Neuron.* 2012;75(1):58–64. 10.1016/j.neuron.2012.04.038 22794260

[76] Enomoto K Matsumoto N Nakai S: Dopamine neurons learn to encode the long-term value of multiple future rewards. *Proc Natl Acad Sci U S A.* 2011;108(37):15462–7. 10.1073/pnas.1014457108 21896766 PMC3174584

[77] Berke JD: What does dopamine mean? *Nat Neurosci.* 2018;21(6):787–93. 10.1038/s41593-018-0152-y 29760524 PMC6358212

[78] Lee RS Mattar MG Parker NF: Reward prediction error does not explain movement selectivity in DMS-projecting dopamine neurons. *eLife.* 2019;8: pii: e42992. 10.7554/eLife.42992 30946008 PMC6464606

[79] Gershman SJ: Dopamine ramps are a consequence of reward prediction errors. *Neural Comput.* 2014;26(3):467–71. 10.1162/NECO_a_00559 24320851

[80] Kim HR Uchida N: A derivative-like computations underlie dopamine prediction error coding based on dynamic sensory stimuli. *Soc Neurosci Abstract.* 2018;109:9.

[81] Lloyd K Dayan P: Tamping Ramping: Algorithmic, Implementational, and Computational Explanations of Phasic Dopamine Signals in the Accumbens. *PLoS Comput Biol.* 2015;11(12):e1004622. 10.1371/journal.pcbi.1004622 26699940 PMC4689534

[82] Schultz W Apicella P Ljungberg T: Responses of monkey dopamine neurons to reward and conditioned stimuli during successive steps of learning a delayed response task. *J Neurosci.* 1993;13(3):900–13. 10.1523/JNEUROSCI.13-03-00900.1993 8441015 PMC6576600

[83] Schultz W Apicella P Ljungberg T: Reward-related activity in the monkey striatum and substantia nigra. *Prog Brain Res.* 1993;99:227–35. 10.1016/s0079-6123(08)61349-7 8108550

[84] Vazey EM Moorman DE Aston-Jones G: Phasic locus coeruleus activity regulates cortical encoding of salience information. *Proc Natl Acad Sci U S A.* 2018;115(40):E9439–E9448. 10.1073/pnas.1803716115 30232259 PMC6176602

[85] Cohen JY Amoroso MW Uchida N: Serotonergic neurons signal reward and punishment on multiple timescales. *eLife.* 2015;4:e06346. 10.7554/eLife.06346 25714923 PMC4389268

[86] Miyazaki K Miyazaki KW Yamanaka A: Reward probability and timing uncertainty alter the effect of dorsal raphe serotonin neurons on patience. *Nat Commun.* 2018;9(1):2048. 10.1038/s41467-018-04496-y 29858574 PMC5984631

